# Nanorings to Probe Mechanical Stress of Single-Stranded DNA Mediated by the DNA Duplex

**DOI:** 10.3390/ijms232112916

**Published:** 2022-10-26

**Authors:** Karen Zagorski, Tommy Stormberg, Mohtadin Hashemi, Anatoly B. Kolomeisky, Yuri L. Lyubchenko

**Affiliations:** 1 Department of Pharmaceutical Sciences, University of Nebraska Medical Center, Omaha, NE 68198, USA; 2 Department of Chemistry, Rice University, 6100 Main Street, Houston, TX 77005, USA

**Keywords:** DNA rings, mechanics of DNA, atomic force microscopy (AFM), time-lapse AFM

## Abstract

The interplay between the mechanical properties of double-stranded and single-stranded DNA is a phenomenon that contributes to various genetic processes in which both types of DNA structures coexist. Highly stiff DNA duplexes can stretch single-stranded DNA (ssDNA) segments between the duplexes in a topologically constrained domain. To evaluate such an effect, we designed short DNA nanorings in which a DNA duplex with 160 bp is connected by a 30 nt single-stranded DNA segment. The stretching effect of the duplex in such a DNA construct can lead to the elongation of ssDNA, and this effect can be measured directly using atomic force microscopy (AFM) imaging. In AFM images of the nanorings, the ssDNA regions were identified, and the end-to-end distance of ssDNA was measured. The data revealed a stretching of the ssDNA segment with a median end-to-end distance which was 16% higher compared with the control. These data are in line with theoretical estimates of the stretching of ssDNA by the rigid DNA duplex holding the ssDNA segment within the nanoring construct. Time-lapse AFM data revealed substantial dynamics of the DNA rings, allowing for the formation of transient crossed nanoring formations with end-to-end distances as much as 30% larger than those of the longer-lived morphologies. The generated nanorings are an attractive model system for investigation of the effects of mechanical stretching of ssDNA on its biochemical properties, including interaction with proteins.

## 1. Introduction

During various biological processes, such as transcription, replication, and packaging, DNA undergoes substantial structural changes. Conformational changes of the DNA duplex have been identified in these processes and are well-known [1,2,3]. At the same time, these processes are accompanied by the transient formation of single-stranded DNA (ssDNA) segments, which coexist with DNA duplex conformations [4,5]. The mechanical properties of both types of DNA are dramatically different. A DNA duplex is a very stiff polymer with a persistence length as large as 150 bp [6], whereas ssDNA is much softer with a persistence length as small as 2–5 residues [7,8].

DNA transcription, replication, repair, and recombination are accomplished by specialized protein machines which produce variations of the DNA duplex structure, such as twisting and bending. Several enzyme families (topoisomerases, gyrases, and helicases) participate in the generation and relaxation of torsional mechanical stress on DNA [9,10]. However, mechanical stress, due to the large persistence length of the DNA duplex, covers large DNA segments. Neighboring soft ssDNA segments can absorb mechanical stress, decreasing the tensions within the DNA duplex. The importance of mechanical stress is clear; DNA polymerases have been shown to display elevated activity at low force [11], while the binding of single-stranded binding proteins such as RPA display force-dependent activity [12]. Moreover, CRISPR/Cas9 off-target activity is increased due to DNA stretching [13]. This interplay in the mechanical properties of ssDNA and the duplex, however, has not been well studied. 

Here, we describe an approach that allowed us to demonstrate how the high stiffness of the DNA duplex induces a mechanical strain on the ssDNA segment. In our approach, a DNA duplex with the size comparable to the persistence length is linked by a ssDNA with 30 residues and connected into a circle, which we refer to as a nanoring. We used atomic force microscopy (AFM) to visualize such hybrid DNA constructs and characterize their structural features. AFM is a powerful single-molecule technique capable of providing conformational details of individual biomolecules with nanometer resolution, as demonstrated by us (e.g., [14] or [15]) and others (reviewed in [16]). Here, we show that tension within the duplex stretches the ssDNA connector, so its end-to-end distance is increased by 16% on average. Time-lapse AFM imaging revealed broad dynamics of the overall shape of such DNA nanorings, capable of significantly extended end-to-end distances, working as unfolding nanotweezers. Theoretical estimates of ssDNA stretching, within the nanoring construct, corroborate the stretching observed in experiments. 

## 2. Results

### 2.1. DNA Nanoring Design

**Figure 1:**
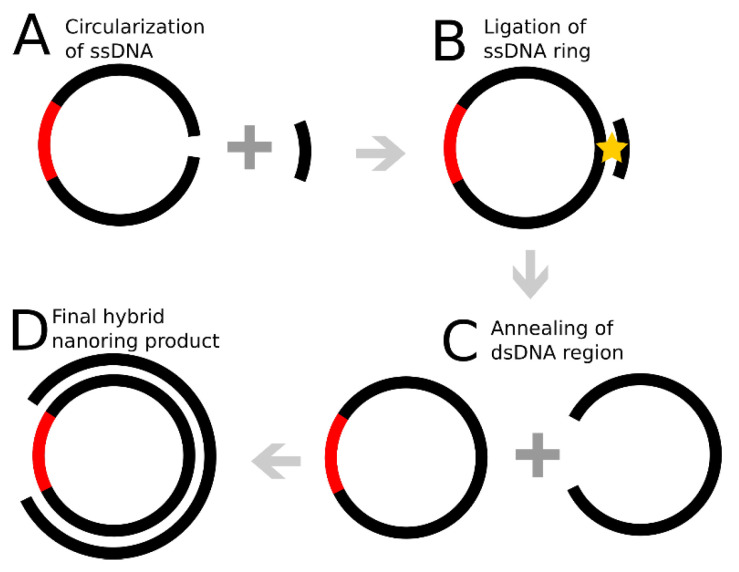
Nanoring circularization. (**A**) The ligation of the 190 nt ssDNA using a splinting oligonucleotide. (**B**) Ligation of the ring with T4 ligase. (**C**) Annealing of the 190 nt ring with the 160 nt ssDNA complement to yield the final ring construct, (**D**), in which 160 bp DNA duplex is connected through the 30 nt ssDNA segment marked with red.

The nanorings were obtained using a multistep process, schematically illustrated in Figure 1. In step one, 190 nt ssDNA was ligated into a ring using a template strand complementary to the ends of the DNA molecule. In step two, the ring was annealed to a 160 nt DNA complement, resulting in the formation of a hybrid DNA circle with 160 bp dsDNA segment connected through a 30 nt ssDNA connector. The final products were analyzed by AFM. 

**Figure 2:**
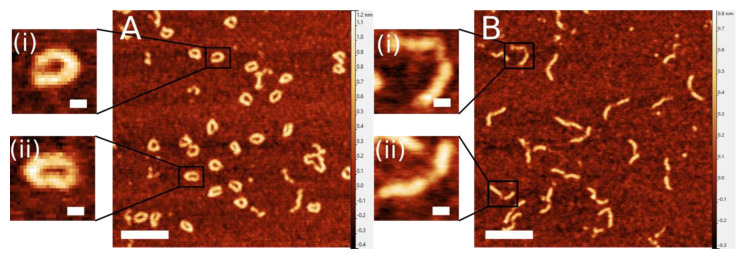
Representative images of nanoring complexes (**A**) and gap DNA constructs (**B**) with zoomed-in examples (**i**,**ii**) imaged in air. Scale bars indicate 100 nm in large-scale images and 10 nm in zoomed-in images.

DNA nanoring samples were deposited on mica functionalized with 1-(3-aminopropyl)- silatrane (APS) [17], incubated for two minutes, dried with a gentle flow of argon, and imaged in air as described in the Methods section. Representative images of these assemblies can be seen in Figure 2A. DNA nanorings appear as circular “c”-shaped molecules in the images. DNA duplex appeared with a high contrast, while ssDNA segment does not show such a high contrast, which is typical for AFM images of ssDNA. A few zoomed-in images of the nanorings are shown in Figure 2A(i,ii). The yield of fully intact DNA nanorings, assessed by the circularity of the molecules, was 82%. 

As a control, we assembled linear DNA constructs, with identical sequence, in which 80 bp DNA duplexes are flanking the same 30 nt ssDNA molecule; we named this construct gap DNA. AFM images of these constructs are shown in Figure 2B. Fully intact gap DNA constructs, possessing both the double-stranded and single-stranded DNA regions, is the dominant species of nanostructures, as seen in Figure 2B. ssDNA segments are clearly seen in the zoomed-in images shown in Figure 2B(i,ii). Gap DNA nanostructures take on a variety of conformations, from those with almost no discernible ssDNA to those with extended ssDNA flanked by duplex DNA oriented at different angles.

### 2.2. Characterization of the Nanorings

**Figure 3:**
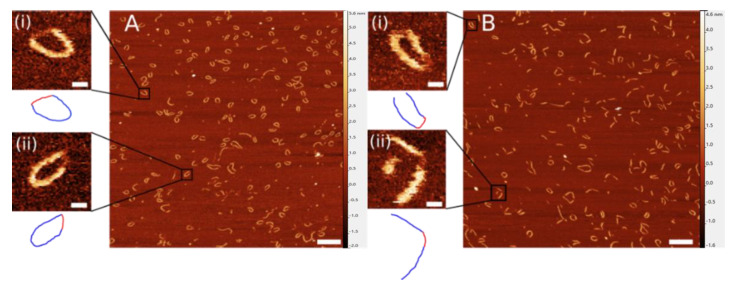
Representative images of nanoring complexes (**A**) and gap constructs (**B**) with zoomed-in examples (**i**,**ii**) imaged in liquid. Cartoons below images depict measurements of double-stranded DNA (blue) and single-stranded DNA (red). Scale bars indicate 100 nm in large-scale images and 10 nm in zoomed-in images.

To improve the accuracy of the DNA length measurements and eliminate potential effect of the sample drying process, we imaged nanorings and gap DNA samples in aqueous solution. Representative images of the DNA nanoring construct and gap DNA construct in aqueous buffer are shown in Figure 3. The blue lines in the traces below the zoomed-in images represent the contours of the dsDNA, while the red lines represent the end-to-end (ETE) distance of the ssDNA. Note that, in the images, we are not able to visualize the path of single-stranded DNA regions primarily due to the low height of ssDNA and its high flexibility. 

**Figure 4:**
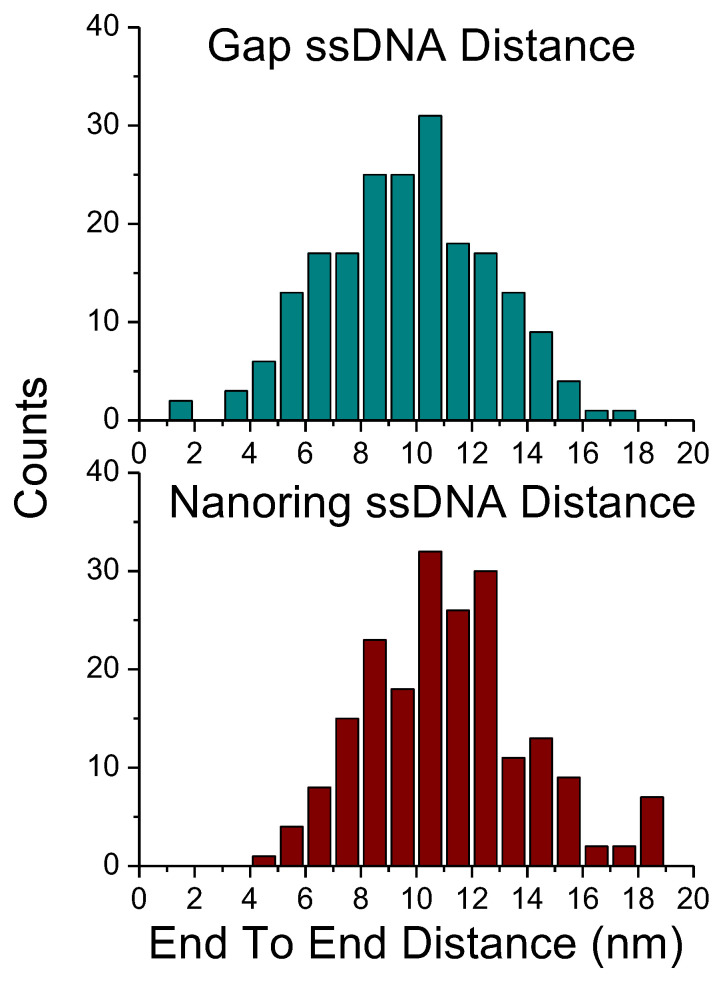
Histograms of end-to-end distance of gap ssDNA and nanoring ssDNA. Statistical comparison of end-to-end distances show a significant increase in nanoring construct compared with the gap construct (*p* < 0.001).

Quantitative analysis of the ETE distance for the nanorings was performed. Figure 4 (bottom) shows the histogram of nanoring ETE distances. Similar measurements were completed for gap DNA constructs and a corresponding histogram is shown in Figure 4 (top). The data show that the mean ETE distance of the nanoring constructs (11.1 nm ± 0.2 SEM, *n* = 265) was 16% larger than the ETE distance of the non-circular gap DNA constructs (9.6 nm ± 0.2 SEM, *n* = 207), indicating a statistically significant stretching effect for ssDNA (*p* < 0.001).

### 2.3. Dynamics of the Nanorings

**Figure 5:**
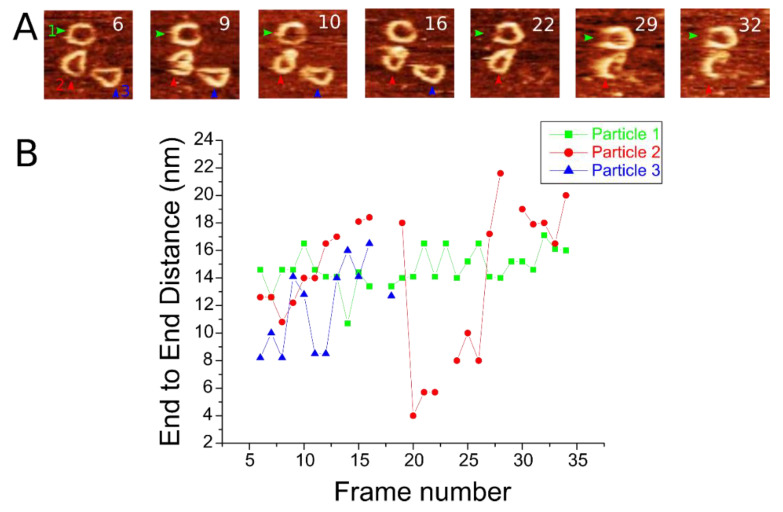
Time-lapse AFM data of nanoring dynamics. (**A**) Selected frames taken from time-lapse experiments following three nanorings. Green arrow indicates particle 1. Red arrow indicates particle 2. Blue arrow indicates particle 3. (**B**) Graph showing the fluctuation of end-to-end distance for each nanoring dependent on the frame number.

Time-lapse AFM imaging of the DNA nanorings allowed us to track the dynamics of individual particles over time. Selective frames from 35 frames of the time-lapse AFM experiment with three individual nanorings are presented in Figure 5A. The full set of the frames is shown in the supplement (Figure 2). Green, red, and blue arrows in each frame point to selected nanorings. The nanoring indicated by the green arrow is shown to occupy roughly the same position across frames. However, a clear change in orientation happens between frames 16 and 22. The single-stranded region, on the right side of the nanoring in frames6–16, is shown to suddenly face left by either “turning” or “flipping” in frame 22. The nanoring indicated by the blue arrow shows fluctuations in end-to-end dsDNA distance before leaving the frame in frame 17, indicative of the freedom of movement of the DNA on the functionalized surface. The nanoring indicated by the red arrow shows the most interesting dynamics; in frame 6, it is shown in a standard circularized shape. However, in frame 9, the nanoring adopts a transient folded shape, similar in shape to a “3”. This shape rapidly changes to a figure “8” shape in frame 10. These transient morphologies display twists and a substantial strain imposed on the ring compared with the circular shaped counterparts, as indicated by the end-to-end distance. This figure “8” persists until frame 22, at which time the nanoring again adopts a circular shape. Over the last ten frames, the ring displays an increased end-to-end difference.

To quantitatively characterize the dynamics of nanorings, we measured the ETE distances for each molecule in Figure 5A and plotted these values in Figure 5B. The green squares, red circles, and blue triangles (colors correspond to Figure 5A) show the fluctuations in particles 1, 2, and 3, respectively. Particle 1 (green arrow) maintained a relatively stable ETE distance throughout the experiment, despite the nanoring “flipping” over, with end-to-end distances predominantly in the range of 13–17 nm. Particle 2 exhibits a larger range, from a more “compact” form in frame 6 corresponding to a distance from 8 nm to as high as 16 nm, before leaving the viewing frame, indicating a large strain upon the nanoparticle. Particle 3 has the most significant fluctuations. After adopting the figure “8” morphology, the ring is more strained over time until frame 19, at which time it rapidly adopts a compact form with end-to-end distances as small as 4 nm. Through the rest of the experiment, the ETE distance increases, reaching a distance of over at one point 20 nm. These transient structures had ETE distances as great as 60% longer than the median for the long-lived morphologies observed in AFM images in air. However, the nanoring remains intact, as it reverts to the standard nanoring shape most commonly seen in frame 22, albeit with significant fluctuations in end-to-end distance. 

### 2.4. Theory of the DNA Nanoring Mechanics

Let us develop a theoretical model for the properties of DNA nanoring molecule that consists of dsDNA segment of length *L* and ssDNA segment of length *l* coupled together in a ring. We can view the ds segment as a part of the circle with the radius *R* and the angle *φ*. The ssDNA segment is viewed as a straight spring. From these geometric arguments we can write that

(1) L=Rφ

and

(2) $$\sin\left(\pi -\frac{\varphi }{2}\right)=\frac{l}{2R}$$

From these equations, we obtain that

(3) $$\varphi =2\pi -2\arcsin\left(\frac{l}{2R}\right)$$

and the circumference is given by

(4) $$2R\arcsin\left(\frac{l}{2R}\right)+L=2\pi R$$

This is an important relation because it allows us to obtain a connection between the radius of the nanoring molecule and the ssDNA segment length *l*.

Now, we estimate the free energy of the nanoring molecule, as follows (in units of *kT*):

(5) $$F_{ring}=\frac{L}{2\pi R}\frac{A}{2\pi R}+\frac{1}{2}k{\left(l-l_{0}\right)}^{2}$$

where *A* is the bending energy parameter, *k* is the spring constant of the ssDNA segment (in units of kT), and *l_0_* is the distance for the relaxed ssDNA spring. We can explain Equation (5) in the following way. The first term describes the bending energy of the dsDNA segment. If we would have a dsDNA loop of length 2πR, then the bending free energy would be equal to $\frac{A}{2\pi R}$, with

(6) $$A=2\pi^{2}L_{p}$$

and *L_p_* is the persistence length, 150 nt~50 nm for dsDNA. However, we only have the part of the loop, and the fraction of the total loop is $\frac{L}{2\pi R}$. The second term in Equation (5) is the spring energy that appears due to the dsDNA stretching of the ssDNA segment.

The experiments also considered the same ss/dsDNA molecules that are not coupled in a ring (gap constructs). For such molecules, we can write the free energy as having only the spring energy contribution, as follows:

(7) $$F_{free}=\frac{1}{2}k{\left(l-l_{0}\right)}^{2}$$

Experiments measured the distribution of distances *l* (ssDNA segment length). In our theoretical framework, they can be computed as follows:

(8) $$P_{free}\left(l\right)=\frac{le^{-F_{free}}}{\int_{0}^{\infty }le^{-F_{free}}dl}$$

(9) $$P_{ring}\left(l\right)=\frac{le^{-F_{ring}}}{\int_{0}^{\infty }le^{-F_{ring}}dl}$$

For free gap constructs, we can even obtain an approximate analytic expression, as follows:

(10) $$P_{free}\left(l\right)\approx \frac{le^{-\frac{1}{2}k{\left(l-l_{0}\right)}^{2}}}{\frac{1}{k}e^{-\frac{1}{2}kl_{0}{}^{2}}+\frac{l_{0}\sqrt{\pi }}{2\sqrt{k}}}$$

Importantly, we predict a Gaussian dependence, and fitting the experimental results will provide the estimate for *l*_0_ and *k*.

Although we can numerically estimate the distributions of gap lengths for both free and nanoring molecules, it is more convenient to consider the most probable gap lengths, *l_min_*, which can be obtained from the experimental measurements. For this purpose, we will use the following series expansion of the arcsin function:

(11) $$\arcsin\left(x\right)≅x+\frac{x^{3}}{6}+\ldots$$

This result suggests that, for *x* up to ~0.8, the first term in Taylor expansion provides a reasonable approximation. Utilizing this in Equation (4) leads to

(12) $$R=\frac{L+l}{2\pi }$$

In experiments, *L* = 160 nt and *l* = 30 nt; then, we estimate that *R*~30 nt and *l*/2*R*~1/2, justifying the approximation.

In this approximation, the free energy of the nanoring system can be written as

(13) $$F_{ring}=\frac{2\pi^{2}LL_{p}}{{\left(L+l\right)}^{2}}+\frac{1}{2}k{\left(l-l_{0}\right)}^{2}$$

Minimizing the free energy with respect to the gap length *l* will estimate the most probable gap distance, as follows:

(14) $$\frac{dF}{dl}=0,\;\mathrm{at}\;l=l_{min}$$

For the free gap constructs we have

(15) $$l_{min}\left(free\right)=l_{0}$$

However, for the nanoring molecules from Equation (13), we obtain (with additional assumption that *l*/*L* << 1)

(16) $$l_{min}\left(ring\right)\approx l_{0}+\frac{4\pi^{2}L_{p}}{kL^{2}}$$

This equation predicts that the increase in the gap length is inversely proportional to the square of dsDNA segment length.

We can also perform the explicit calculations by considering the known result from the polymer that the spring constant for ssDNA is given by

(17) $$k=\frac{3\;kT}{NL_{k}}$$

where *L_k_* is a Kuhn length (*L_k_* = 3 nm for ssDNA) and *N* is the number of Kuhn segments in the ssDNA segment (*N* = 3 for 30 nt segment). This leads to the following result (all lengths are in nm):

(18) $$l_{min}\left(ring\right)\approx l_{0}+\frac{6000}{L^{2}}$$

For the experimental system with *L* = 160 nt = 53 nm, we find that the increase in the gap length is ~2.3 nm. This estimate is fully consistent with experimental data on the difference in lengths *l_min_*~9.5 nm for free molecules and *l_min_*~11.5 nm for nanorings.

## 3. Discussion

In this paper, we investigated a mechanical interplay between rigid dsDNA and softer ssDNA. To accomplish this goal, we developed and used a hybrid DNA approach in which ssDNA segment was inserted inside the DNA duplex to from a nanoring. Our results revealed several interesting features of such a construct.

We used the DNA nanoring with the DNA duplex part with the size comparable with the DNA persistence length [6,18] and ssDNA with 30 residues to form a nanoring with a diameter ~20 nm. These molecules were imaged with AFM, and DNA duplexes and ssDNA segments were visualized (Figure 2). Analysis revealed several important properties of ssDNA in such constructs. We have shown that ssDNA within nanoring is extended by 16% compared to the linear construct, in which ssDNA is not under stress (Figure 4). Time-lapse AFM imaging (Figure 5) demonstrated that the ssDNA is quite dynamic, collapsing to a coil with the distance ~4 nm followed by 5-fold extension, producing an end-to-end distance as large as ~20 nm.

A theoretical model built on the known mechanical properties of ssDNA and duplexes associates ssDNA extension in nanorings with a force of ~4 pN. Such a force is comparable to the force induced by a thermal motion, which explains the high dynamics of the DNA nanorings visualized by the time-lapse AFM experiments. The DNA duplex can form a supercoil (frame 9 in Figure 5A) with a compact conformation of ssDNA; the supercoil dissociation eventually leads to stretching ssDNA to values as large as 20 nm (frame 32 in Figure 5A).

Mechanical force is a critical physiological factor involved in a variety of biological processes. Primarily high forces are produced during actions of cellular machines such as DNA and RNA polymerases [19]. Since DNA in chromosomes can be topologically constrained, forces produced by molecular machines can be propagated to ssDNA segments leading to their extension. ssDNA is a target for binding a number of proteins, including SSB protein of bacteria or RPA protein for eukaryotes. Extension of ssDNA can change the mode of interaction of such proteins with ssDNA and such effect has been reported for RPA protein [12].

We would like to emphasize that the property of the hybrid nanoring approach could be used as a nanotool for generating forces. The current construct with the DNA duplex of 160 bp and the ssDNA size of 30 nt produces a force of ~4 pN. This value can be increased by decreasing the duplex length, and Equation (16) can be used for estimates of the DNA length in designing the DNA nanoring construct with defined extension force. Importantly, the DNA duplex is under stress in such a construct, resulting in the change of the DNA duplex conformation in hybrid nanorings. Such an interplay between structural mechanical properties of single-stranded and double-stranded DNA can be a factor defining interaction of various DNA-binding proteins and especially those involved in various DNA transactions, including DNA replication, making the hybrid DNA ring approach attractive in such studies.

## 4. Materials and Methods

### 4.1. Oligonucleotides for Nanoring Assembly

The DNA sequence used for the nanoring was designed using data from [18] for ideal DNA rigidity and then optimized for amelioration of hairpin formation. Oligonucleotides of following sequences were acquired commercially from Integrated DNA Technologies (IDT, Coralville, IA) with PAGE purification:

- 190 nt pre-ring oligo: 5′-TCATGGAAGGATATGTACGATATCGAGATCTAGCTATCCCGGCTATGTGCATATCGAGCGCTGTCTACATATGGATACTCCTATGTCACCGGTCTCTAGTCTCGATGATCTTATGTCCTAGTGTCGCCATGCTGTGTGCGGTACCGTGAGACACAGATCGTATGCAGCTATACGGCAGTCCATAGGAGCC-3′
- 160 nt hybridization oligo: 5′-CATATGTAGACAGCGCTCGATATGCACATAGCCGGGATAGCTAGATCTCGATATCGTACATATCCTTCCATGAGGCTCCTATGGACTGCCGTATAGCTGCATACGATCTGTGTCTCACGGTACCGCACACAGCATGGCGACACTAGGACATAAGATCATC-3′
- 20 nt splinting oligo: 5′-AGTCCATAGGAGCCTCATGGAAGG-3′
- 80 nt hybridization oligo 1: 5′-ATGGACTGCCGTATAGCTGCATACGATCTGTGTCTCACGGTACCGCACACAGCATGGCGACACTAGGACATAAGATCATC-3′
- 80 nt hybridization oligo 2: 5′-CATATGTAGACAGCGCTCGATATGCACATAGCCGGGATAGCTAGATCTCGATATCGTACATATCCTTCCATGAGGCTCCT-3′

### 4.2. Preparation of Nanorings

Circularization of 190t ssDNA was performed using a modified version of the protocol published in [20]. The process is shown schematically in Figure 1. Briefly, a 10 μL mixture containing 2 μM 190 nt pre-ring DNA, 1× T4 ligation buffer, and 1× T4 polynucleotide kinase was incubated at 37 °C for 60 min. The resulting DNA was then incubated with the 20 nt splinting oligo at a 1:1.5 DNA:primer ratio at 16 °C for 20 min.

The DNA:primer mixture was then incubated in a 1 mL solution containing 0.1× T4 ligation buffer and 400 units of T4 DNA ligase, resulting in a final concentration ratio of 20 nM DNA:30 nM primer. This mixture was incubated at 16 °C overnight. Fresh 10× T4 ligation buffer was then added to achieve a final 1× buffer concentration, followed by an additional incubation at 4 °C for 3 days to complete ligation.

After ligation was completed, the mixture was incubated at 65 °C for 10 min to inactivate the ligase. A 1.15× excess of 160 nt hybridization oligo was added to the mixture and heat-annealed. Annealing was achieved by suspending the mixture in 400 mL of boiling water which was then left to gradually cool to room temperature. Rings were then stored at 4 °C in buffer containing 10 mM Tris, pH 8.0. A measure of 1 mM EDTA may be added for long-term storage.

### 4.3. Preparation of Gap Constructs

Gap constructs were prepared with a simple annealing step. The 190 nt pre-ring oligo was incubated with 80 nt hybridization oligo 1 and 2 at a ratio of 1:1.15:1.15 pre-ring oligo:hybridization oligo 1:hybridization oligo 2. The mixture was suspended in boiling water that was then allowed to cool to room temperature. The gap constructs were stored at 4 °C in buffer containing 10 mM Tris, pH 8.0.

### 4.4. Static Atomic Force Microscopy Imaging in Air

Freshly cleaved mica was functionalized with a 167 μM solution of 1-(3-aminopropyl)- silatrane (APS) for 30 min at room temperature, rinsed, and dried with a gentle flow of argon as described in [17]. Samples were diluted to 2 nM concentration in imaging buffer (10 mM HEPES pH 7.5, 4 mM MgCl_2_), deposited on the mica surface for 2 min, rinsed with water, and dried with gentle argon flow. Samples were stored in vacuum under argon until ready for imaging using tapping mode on a Nanoscope V MultiMode 8 system (Bruker, Santa Barbara, CA, USA) using TESPA probes with a nominal tip radius of 7 nm (Bruker, Santa Barbara, CA, USA). A typical image was 1 × 1 μm in size with 2 nm/pixel resolution.

### 4.5. Time-Lapse Atomic Force Microscopy Imaging in Aqueous Buffer

Functionalized mica was prepared, and samples were deposited as described above. A measure of 10 mM Tris-HCl pH 8.0 buffer was used for liquid imaging. The AFM used in this work is a Nanoscope V system modified to incorporate a small cantilever head during an open-science workshop at EPFL in the laboratory of Professor Georg Fantner [21]. Samples were imaged using photothermal off-resonance tapping (PORT) [22] using FASTSCAN-D probes (Bruker, Santa Barbara, CA, USA). Captured images were 500 × 500 nm in size at a resolution of 1 nm/pixel.

### 4.6. Image Analysis

The acquired frames were analyzed using FemtoScan software (Advance Technologies Center, Moscow, Russia). The ends of dsDNA regions were identified based on 2 nm height of the dsDNA and the shortest path between the two ends of the dsDNA was used as the equivalent of the end-to-end distance of the ssDNA. The non-circular gap construct was used as a reference for non-strained ssDNA end-to-end distance. We also saved a snapshot of each measured particle to use for duplex-morphology-based characterization and analysis. Finally, we utilized time-lapse AFM to visualize the dynamic behavior of the generated rings.

## Data Availability

All data are included in the manuscript.
